# HCV Genotypes of People with Hemophilia in Countries with High Seroprevalances

**Published:** 2011-02-01

**Authors:** J N Mahlangu, N Prabdial-Sing

**Affiliations:** 1 Haemophiliac Comprehensive Care Center, Charlotte Maxeke, Johannesburg academic Hospital, National Health Laboratory Services and University of the Witwatersrand, Parktown, South Africa; 2 Hepatitis Virology Division, Specialized Molecular Diagnostic Unit, National Institute for Communicable Diseases of the National Health Laboratory Services, Sandringham, South Africa

**Keywords:** Hemophilia, HCV, Genotypes, Mixed infections

Dear Editor

The risk of transfusion-transmitted hepatitis C virus (HCV) infection was the greatest in people with haemophilia (PWH) before identification and testing for this virus became available in 1987. After introduction of HCV screening and virucidal measures post 1987, this risk significantly reduced in the developed countries (1.8%).[1] However, in resource poor settings, the risk still remained high. The HCV seroprevalence rate among PWH in Brazil was as high at 44%,[2] with that in Pakistan higher at 56%.[3]

In the haemophilia population, diagnosis of HCV appears to be earlier (29-31 years),[4] compared to other non-hemophilia patient populations (52 years).[5] This difference could be due to a number of reasons, chronicity of hemophilia and close clinical follow of PWH by dedicated teams being some of the contributing factors. Knowledge on HCV genotypes and viral loads are important in making treatment decisions as well as predicting response to treatment. From a public health perspective, knowledge of the genotype frequencies and their changes can provide important epidemiological information for screening and preventative public programs.[6] HCV genotyping tests have transformed over the years from in-house sequencing to commercialised line probe assays (LiPA) and as well as real-time PCR analyses. However, the method of choice should be sensitive enough to detect all HCV genotypes including mixed genotype infections. Although earlier reports seem to indicate that mixed genotype infections were rare,[7] recent data indicate that the diagnosis of mixed genotypes has been increasing as indicated by Alavian et al.,[8] (4%; 1 and 3, 3 and 4) and Oh et al.,[9] (4.4%, genotype 1b and 2a/c). This is largely as a result of evolution of more sensitive HCV genotyping assays. Consequently, the apparent discrepancy between the mixed genotype rates reported by the two studies done in Iran at different time frames rate 6.2%[4] and 27%[10] that could be ascribed in part to the availability of more sensitive methods.

Efforts to start and continue the diagnosis and treatment of HCV in sentinel groups are needed to reduce transmission of the virus and the future burden of chronic liver diseases. The cost of sensitive and specific PCR and genotyping methods is daunting for countries with limited resources. However, surveillance systems and notification to government is required so that awareness is raised and proper action taken. Public education campaigns like the “start today, tomorrow may be late”[4] and “Be Blood Aware”[1] are good role models to follow. Although total population of PWH screened by Keshvari and colleagues (2010) represent less that 10% of the total hemophilia population in Iran,[4] this effort is to be applauded as it signals a step in the right direction to total management of HCV in PWH.
